# Supplementary material for: Identification of Freezing-Responsive microRNAs and Their Targets in Chinese Jujube by Small RNA and Degradome Sequencing
Source: Int J Mol Sci. 2026 Sep 4;27(17):7912. doi: 10.3390/ijms27177912 (PMC13566890; doi:10.3390/ijms27177912)
Supplement: Supplementary file 1 [file ijms-27-07912-s001.zip › Supplementary Material S2/novel_miR_228.pdf]

Provisional ID	:	novel_miR_228
Score total	:	8308.8
Score for star read(s)	:	3.9
Score for read counts	:	8302.1
Score for mfe	:	1.2
Score for randfold	:	1.6
Score for cons. seed	:	
Total read count	:	16296
Mature read count	:	16098
Loop read count	:	8
Star read count	:	190

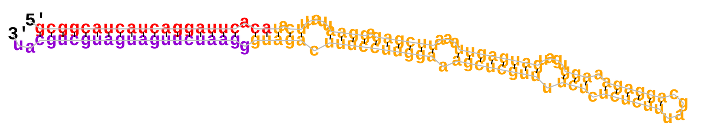

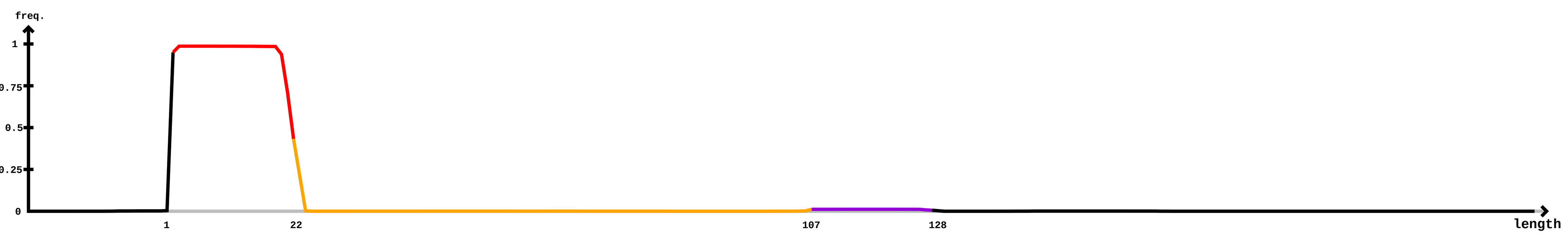

Mature Star

```
5'- ugaucagucaguauuuucggaugcggcaucaucaggauucacauacuuuauuaaggagagcuuaaauugaguaguaguggaaagaggacgauuucucucucuuuugcucgaaagguuccuuucagaugggaaucuugaugaugcugcaucagcaaauauaugacuaugucucccuguuacuucucacauaaauuccaaauauauaaaccccauauauauauauaaaccccauauauauauauauauauau -3'  obs
    ugaucagucaguauuuucggaugcggcaucaucaggauucacauacuuuauuaaggagagcuuaaauugaguaguaguggaaagaggacgauuucucucucuuuugcucgaaagguuccuuucagaugggaaucuugaugaugcugcaucagcaaauauaugacuaugucucccuguuacuucucacauaaauuccaaauauauaaaccccauauauauauauaaaccccauauauauauauauauauau       exp
    .(((.((((((((((.(.((((((((((((((((((((.(((.((.....((((.((((((...((((((((....(((.((((((....)))))).))).)))))))).)))))))))).))))).))))))))))))))))))))).).)))))).)))))..)))..........................(((((((.......((((((((((((.......)))))))))))))))))))
```

	reads	mm	sample
.......ucaguauuuucggaugcggCucauc	1	1	LT-2
.......ucaguauuuucggaugcggcaucauca	1	0	LT-1
...........uauuuucggaugcggcauc	1	0	LT-3
...........uauuuucggaugcggcauc	1	0	LT-1
...........uauuuucggaugcggcaucauc	1	0	LT-1
............auuuucggaugcggcauc	1	0	LT-3
............auuuucggaugcggcaucauc	1	0	LT-3
............auuuucggaugcggcaucauc	1	0	LT-2
.............uuuucggaugcggcaucaucagg	3	0	JT-3
.............uuuucggaugcggcaucaucagg	5	0	JT-2
.............uuuucggaugcggcaucaucagga	2	0	LT-3
.............uuuucggaugcggcaucaucagga	2	0	LT-1
.............uuuucggaugcggcaucaucagga	1	0	LT-2
.............uuuucggaugcggcaucaucaggaA	2	1	JCK-2
.............uuuucggaugcggcaucaucaggaA	2	1	JCK-3
...............uucggaugcggcaucauca	1	0	LT-1
................ucggaugcggcaucaucaggU	1	1	LT-3
................ucggaugcggcaucaucagga	1	0	JCK-3
................ucggaugcggcaucaucaggau	2	0	JCK-3
................ucggaugcggcaucaucaggau	1	0	JCK-2
..................ggaugcggcaucaucaggauu	1	0	JCK-2
.....................Cgcggcaucaucaggauuca	1	1	LT-3
.....................Cgcggcaucaucaggauuca	2	1	LT-1
.....................ugcggcaucaucaggauucac	1	0	JCK-2
.....................ugcggcaucaucaggauucac	1	0	LT-3
.....................ugcggcaucaucaggauucac	2	0	JCK-1
.....................ugcggcaucaucaggauucac	1	0	JCK-3
.....................ugcggcaucaucaggauucaca	3	0	LT-3
.....................Cgcggcaucaucaggauucaca	1	1	LT-3
.....................ugcggcaucaucaggauucacau	2	0	JCK-2
.....................ugcggcaucaucaggauucacau	2	0	JCK-1
.....................ugcggcaucaucaggauucacau	9	0	JT-1
.....................ugcggcaucaucaggauucacauU	2	1	JCK-3

Mature Star

ugaucagucaguauuuucggaugcggcaucaucaggauucacauacuuuauuaaggagagcuuaaauugaguaguaguggaaagaggacgauuucucucucuuuugcucgaaagguuccuuucagauggggaaucuugaugaugcugcaucagcaaauauaugacuaugucucccuguuacuucucacauaaauuccaaauauauaaaccccauauauauauauaaaccccauauauauauauauauauau

ugcggcaucaucaggauucacaua	1	0	JT-2
ugcggcaucaucaggauucacauU	2	1	JCK-2
gcggcaucaucaggauuc	47	0	LT-2
gcggcaucaucaggauuc	67	0	LT-1
gcggcaucaucaggauuA	3	1	LT-1
Acggcaucaucaggauuc	1	1	LT-2
gcggcaucaucaggauuc	129	0	LT-3
gcggcaucaucagNauuc	1	1	LCK-1
gcggcaucaucaggauuc	41	0	JT-1
gcggcaucaucaggauuA	1	1	JCK-3
gcggcaucaucaggauuc	71	0	JCK-3
gcggcaucaucaggauuU	1	1	JCK-2
gcggcaucaucaggauuc	93	0	LCK-3
gcggcaucaucaggauuU	8	1	LT-2
gcggcaucCucaggauuc	1	1	LCK-3
gcggcaucaucaggauuA	2	1	LCK-3
Ucggcaucaucaggauuc	1	1	LT-3
gcggcaucaucaggauuc	37	0	LCK-1
gcggcaucaucaggauuA	1	1	LT-2
Ccggcaucaucaggauuc	1	1	JT-1
gcggcaucaucaggauuA	23	1	JT-1
gcggcaucaucaggauuc	99	0	JCK-2
gcggcaucaucaggauuc	4	0	JT-2
gcggcaucaucaggauuc	68	0	JCK-1
gcggcaucaucaggauuU	1	1	LT-1
gcggcaucaucCggauuc	1	1	JCK-3
gcggcaucaucaggauuU	7	1	LT-3
gcggcaucaucaggauuc	45	0	LCK-2
gcggcaucaucaggauuA	4	1	LT-3
gcggcaucaucaggauuc	6	0	JT-3
gcggcaucCucaggauuca	1	1	LT-3
gcggcaucCucaggauuca	1	1	JCK-1
gcggcaucaucaggauucU	1	1	JCK-1
gcAgcaucaucaggauuca	1	1	LT-2
Acggcaucaucaggauuca	1	1	LCK-2
gcggcaucaucCggauuca	1	1	LT-2
gcggcaucaucaggauuca	157	0	LCK-2
gcggUaucaucaggauuca	1	1	JCK-3
gcggcaucaucaggauuca	604	0	LT-3
gcggcaucaucaggauuca	43	0	JT-3
gcggcaucaucNggauuca	1	1	JCK-1
gcggcaucCucaggauuca	3	1	JCK-2
gcggcaucaucCggauuca	1	1	LT-3
Acggcaucaucaggauuca	2	1	LCK-3
Ucggcaucaucaggauuca	1	1	JT-1
gcggUaucaucaggauuca	1	1	JCK-2
gcggcaucaucaggauucG	1	1	JCK-3
Acggcaucaucaggauuca	2	1	LT-3
gcggcaucaucaggauucU	1	1	LT-1
gcggcaucaucCggauuca	1	1	JCK-2
gcggcaucaucaggauuca	309	0	LT-1
gcggcaucaucaggauuca	474	0	JCK-2
gcggcaucaucaggCuuca	1	1	LT-3
gcggcaucaucCggauuca	1	1	JT-2
gNggcaucaucaggauuca	1	1	JT-1
gcggcaucaucaggauuUa	1	1	LT-1
gcggcaucaucagAauuca	1	1	LT-3
gcgAcaucaucaggauuca	1	1	LT-2
gcggcaucaucaggauuca	21	0	JT-2
gcgAcaucaucaggauuca	1	1	JT-1
gcggcaucaucaggauucU	1	1	LT-3
gcggcaucaucaggCuuca	1	1	LT-2
gcggcaucaucaggauuca	318	0	JCK-1
gcggcaucaucaggCuuca	1	1	LCK-3
gcggcaucaucaggauucC	1	1	JCK-2
gcggcaucaucaggNuuca	2	1	LCK-1
gcggcaucaucaggauuca	288	0	JT-1
gcggcaucaucaggauuca	168	0	LCK-1
gcggcaucaucaggauuca	570	0	LCK-3
gcggcaucaucaggauucC	1	1	LT-3

```
                      Mature                                                                                                    Star
ugaucagucaguauuuucggaugcggcaucaucaggauucacauacuuuauuaaggagagcuuaaauugaguaguaguggaaagaggacgauuucucucucuuuugcucgaaagguuccuuucagaugggaaucuugaugaugcugcaucagcaaauauaugacuaugucucccuguuacuucucacauaaauuccaaauauauaaaccccauauauauauauaaaccccauauauauauauauauau
......................gcggcaucaucagNauuca...............................................................................................................................................................................................................   2      1   LCK-1
......................gcggcaucaucCggauuca...............................................................................................................................................................................................................   1      1   JT-1
......................gcggcaucaucaggauuca...............................................................................................................................................................................................................   358    0   LT-2
......................gcggcaucaucaAgauuca...............................................................................................................................................................................................................   1      1   JT-1
......................gcggcaucCucaggauuca...............................................................................................................................................................................................................   1      1   LT-2
......................gcggcaucaucaggauuca...............................................................................................................................................................................................................   338    0   JCK-3
......................Ucggcaucaucaggauuca...............................................................................................................................................................................................................   1      1   LT-2
......................gNggcaucaucaggauuca...............................................................................................................................................................................................................   1      1   LT-2
......................gcggcaucaucaggCuuca...............................................................................................................................................................................................................   1      1   LCK-1
......................gcggcaucaucCggauuca...............................................................................................................................................................................................................   1      1   JCK-1
......................gcggcaucaucCggauucac..............................................................................................................................................................................................................   1      1   LT-3
......................gcgAcaucaucaggauucac..............................................................................................................................................................................................................   1      1   JCK-3
......................gcggcaucaucaggauucac..............................................................................................................................................................................................................   27     0   LCK-2
......................gNggcaucaucaggauucac..............................................................................................................................................................................................................   2      1   JCK-1
......................gcggcaucaucaggGuucac..............................................................................................................................................................................................................   1      1   JCK-3
......................gcggcaucaucNggauucac..............................................................................................................................................................................................................   1      1   JT-1
......................gcggcaucaucaggCuucac..............................................................................................................................................................................................................   2      1   JCK-2
......................gcgCcaucaucaggauucac..............................................................................................................................................................................................................   1      1   JCK-3
......................gcAgcaucaucaggauucac..............................................................................................................................................................................................................   1      1   JCK-2
......................Acggcaucaucaggauucac..............................................................................................................................................................................................................   1      1   JCK-2
......................Acggcaucaucaggauucac..............................................................................................................................................................................................................   2      1   LCK-3
......................gcggcaucaucNggauucac..............................................................................................................................................................................................................   2      1   JCK-1
......................gNggcaucaucaggauucac..............................................................................................................................................................................................................   1      1   JCK-2
......................gcggcaucaucaggCuucac..............................................................................................................................................................................................................   1      1   JCK-3
......................gcggcCucaucaggauucac..............................................................................................................................................................................................................   2      1   JCK-2
......................gcggcaucaucaggauucac..............................................................................................................................................................................................................   224    0   LT-2
......................Acggcaucaucaggauucac..............................................................................................................................................................................................................   2      1   LT-1
......................gcggUaucaucaggauucac..............................................................................................................................................................................................................   1      1   LT-2
......................gcAgcaucaucaggauucac..............................................................................................................................................................................................................   1      1   JCK-1
......................gcggcaucaucaggGuucac..............................................................................................................................................................................................................   1      1   JCK-2
......................gcggcaucaucaggCuucac..............................................................................................................................................................................................................   1      1   JCK-1
......................gcggcaucaucaggauucac..............................................................................................................................................................................................................   953    0   JCK-3
......................gcggcaucaucaggauucac..............................................................................................................................................................................................................   444    0   LT-3
......................gcggcaucaucaggauuGac..............................................................................................................................................................................................................   1      1   JCK-3
......................gcggcaucaucCggauucac..............................................................................................................................................................................................................   1      1   JCK-1
......................gcggcaucaucaggauucaA..............................................................................................................................................................................................................   2      1   LT-1
......................gcggcaucaucaggauucaA..............................................................................................................................................................................................................   12     1   JCK-3
......................gcggcaucaucaggaCucac..............................................................................................................................................................................................................   1      1   LCK-3
......................gcggcaucaucaggauucaN..............................................................................................................................................................................................................   2      1   JCK-1
......................gcggcaucaucaggauucaU..............................................................................................................................................................................................................   11     1   JCK-2
......................gcggcaucaucaggauucaU..............................................................................................................................................................................................................   8      1   JCK-1
......................gcggcaucaucaggauucaA..............................................................................................................................................................................................................   10     1   JCK-2
......................gcggcaucaucaggauucaU..............................................................................................................................................................................................................   3      1   LT-1
......................gcggcaucaucaggauucCc..............................................................................................................................................................................................................   2      1   JCK-1
......................gcggcaucaucaggauucaU..............................................................................................................................................................................................................   10     1   LCK-3
......................gcgAcaucaucaggauucac..............................................................................................................................................................................................................   1      1   LCK-3
......................gcggcaucaucaggauCcac..............................................................................................................................................................................................................   1      1   JCK-2
......................gcggcaucaucaggauucCc..............................................................................................................................................................................................................   1      1   JCK-2
......................gcggcaucCucaggauucac..............................................................................................................................................................................................................   3      1   JCK-2
......................gcggcaucauAaggauucac..............................................................................................................................................................................................................   1      1   JCK-3
......................gcggcaucaucaggauucaA..............................................................................................................................................................................................................   3      1   JCK-1
......................gcggcaucaucaggauucac..............................................................................................................................................................................................................   20     0   LCK-1
......................gcggcaucaucaggauucac..............................................................................................................................................................................................................   319    0   LT-1
......................gcggcaucaucaggauucac..............................................................................................................................................................................................................   1298   0   JCK-2
......................gcggcGucaucaggauucac..............................................................................................................................................................................................................   1      1   JCK-2
......................gcggcaucCucaggauucac..............................................................................................................................................................................................................   1      1   JCK-1
......................gcggcaucaucCggauucac..............................................................................................................................................................................................................   1      1   JCK-2
......................gcggcaucaucaggauucac..............................................................................................................................................................................................................   30     0   JT-2
......................gcggcaucaucaggauucac..............................................................................................................................................................................................................   780    0   JCK-1
......................gcggcaucaucaggauucac..............................................................................................................................................................................................................   115    0   JT-1
......................gcggUaucaucaggauucac..............................................................................................................................................................................................................   1      1   LT-3
......................Acggcaucaucaggauucac..............................................................................................................................................................................................................   2      1   JCK-1
......................gcgAcaucaucaggauucac..............................................................................................................................................................................................................   1      1   JCK-2
......................gcggcaucaucaggauucaU..............................................................................................................................................................................................................   5      1   LT-3
......................Ucggcaucaucaggauucac..............................................................................................................................................................................................................   1      1   LT-1
......................Ucggcaucaucaggauucac..............................................................................................................................................................................................................   1      1   LCK-2
......................gcggcCucaucaggauucac..............................................................................................................................................................................................................   1      1   LT-3
......................gcggcaucaucaggauucac..............................................................................................................................................................................................................   122    0   LCK-3
......................gcggcaucaucaggauucaA..............................................................................................................................................................................................................   2      1   LT-3
......................gcggcaucaucaggauucaU..............................................................................................................................................................................................................   17     1   JCK-3
```

```
                      Mature                                                                                                      Star
ugaucagucaguauuuucggaugcggcaucaucaggauucacauacuuuauuaaggagagcuuaaauugaguaguaguggaaagaggacgauuucucucucuuuugcucgaaagguuccuuucagaugggaaucuugaugaugcugcaucagcaaauauaugacuaugucucccuguuacuucucacauaaauuccaaauauauaaaccccauauauauauauaaaccccauauauauauauauauau
......................gcggcCucaucaggauucac..............................................................................................................................................................................................  2    1   JCK-1
......................gcggcaucaucaggauucac..............................................................................................................................................................................................  30   0   JT-3
......................gcggcaucaucaggauucacU.............................................................................................................................................................................................  3    1   LT-2
......................Acggcaucaucaggauucaca.............................................................................................................................................................................................  2    1   LT-3
......................gcggcaucaucaggauucacC.............................................................................................................................................................................................  2    1   JCK-3
......................gcggcaucaucaggauucacU.............................................................................................................................................................................................  4    1   LT-1
......................gcggcaucaucaggauucacC.............................................................................................................................................................................................  1    1   JT-1
......................gNggcaucaucaggauucaca.............................................................................................................................................................................................  1    1   JT-1
......................gcggcaucaucaggauucacC.............................................................................................................................................................................................  1    1   JCK-1
......................gcggcaucaucaggauucacC.............................................................................................................................................................................................  3    1   LT-2
......................gcggcaucaucaggauucaca.............................................................................................................................................................................................  485  0   LT-1
......................gcggcCucaucaggauucaca.............................................................................................................................................................................................  1    1   LT-2
......................gcggcaucCucaggauucaca.............................................................................................................................................................................................  1    1   JCK-1
......................gcggcaucaucaggauucaca.............................................................................................................................................................................................  807  0   JCK-1
......................gcUgcaucaucaggauucaca.............................................................................................................................................................................................  1    1   JT-1
......................Acggcaucaucaggauucaca.............................................................................................................................................................................................  1    1   JCK-3
......................gcggcaucaucaggauucaca.............................................................................................................................................................................................  582  0   LT-2
......................gcggcaucaucCggauucaca.............................................................................................................................................................................................  2    1   JCK-2
......................gcggcaucaucaggaCucaca.............................................................................................................................................................................................  1    1   JT-1
......................gcggcaucaucNggauucaca.............................................................................................................................................................................................  1    1   JT-1
......................gcggcaucaucaggauucacU.............................................................................................................................................................................................  8    1   LT-3
......................gcggcaucaucaggauucaca.............................................................................................................................................................................................  363  0   LCK-3
......................gcggcaucaucaggauucaca.............................................................................................................................................................................................  742  0   JT-1
......................Ucggcaucaucaggauucaca.............................................................................................................................................................................................  1    1   LT-3
......................gcggcaucaucaggauucaca.............................................................................................................................................................................................  724  0   LT-3
......................gcggcaucaucaggauucaAa.............................................................................................................................................................................................  1    1   LT-2
......................gcggcaucaucNggauucaca.............................................................................................................................................................................................  1    1   JCK-1
......................gcggcaucaucaggauucCca.............................................................................................................................................................................................  1    1   LT-2
......................gcAgcaucaucaggauucaca.............................................................................................................................................................................................  1    1   JCK-1
......................gcggcaucCucaggauucaca.............................................................................................................................................................................................  1    1   LCK-3
......................gcgAcaucaucaggauucaca.............................................................................................................................................................................................  1    1   JCK-2
......................gcggcaucaucaggauucaAa.............................................................................................................................................................................................  3    1   JCK-3
......................gcggcaucaucaggauucaca.............................................................................................................................................................................................  78   0   JT-3
......................gcggcaucaucaggCuucaca.............................................................................................................................................................................................  2    1   LT-2
......................Ccggcaucaucaggauucaca.............................................................................................................................................................................................  1    1   LT-2
......................gcggcaucaucagAauucaca.............................................................................................................................................................................................  1    1   LT-3
......................Ucggcaucaucaggauucaca.............................................................................................................................................................................................  1    1   JT-3
......................gcggcaucaucaggauucaca.............................................................................................................................................................................................  66   0   LCK-1
......................gNggcaucaucaggauucaca.............................................................................................................................................................................................  1    1   JCK-1
......................gcAgcaucaucaggauucaca.............................................................................................................................................................................................  1    1   JCK-3
......................gcggcaucaucGggauucaca.............................................................................................................................................................................................  1    1   JCK-3
......................gcggcaucaucaggauucCca.............................................................................................................................................................................................  1    1   LT-3
......................Acggcaucaucaggauucaca.............................................................................................................................................................................................  2    1   LT-1
......................gcggUaucaucaggauucaca.............................................................................................................................................................................................  1    1   JCK-3
......................gcggcaucaucaggGuucaca.............................................................................................................................................................................................  2    1   LT-3
......................gcggcaucaucaggauucCca.............................................................................................................................................................................................  1    1   LT-1
......................gcggcaucCucaggauucaca.............................................................................................................................................................................................  2    1   JT-1
......................gcggcaucaucaggauucaca.............................................................................................................................................................................................  952  0   JCK-3
......................gcggcaCcaucaggauucaca.............................................................................................................................................................................................  1    1   LT-3
......................Acggcaucaucaggauucaca.............................................................................................................................................................................................  1    1   JT-1
......................gcggcaucaucaggauucaca.............................................................................................................................................................................................  66   0   JT-2
......................Acggcaucaucaggauucaca.............................................................................................................................................................................................  2    1   JCK-2
......................gcggcCucaucaggauucaca.............................................................................................................................................................................................  1    1   LT-1
......................gcggcaucaucaggauucacN.............................................................................................................................................................................................  1    1   JCK-1
......................gcggcaucaucaggauucacG.............................................................................................................................................................................................  2    1   JCK-2
......................gcggcaucaucaggauucacU.............................................................................................................................................................................................  9    1   JCK-2
......................gcggcGucaucaggauucaca.............................................................................................................................................................................................  1    1   LT-1
......................gcggcaucaucaggauucCca.............................................................................................................................................................................................  1    1   JCK-2
......................Acggcaucaucaggauucaca.............................................................................................................................................................................................  1    1   LT-2
......................gcggcaucaucaggauucacU.............................................................................................................................................................................................  4    1   JCK-1
......................gcggcaucaucaUgauucaca.............................................................................................................................................................................................  1    1   JCK-3
......................gcggcaucaucaggauucaUa.............................................................................................................................................................................................  2    1   JCK-3
......................gcggUaucaucaggauucaca.............................................................................................................................................................................................  1    1   JT-3
......................gcggcaucaucaggauucacU.............................................................................................................................................................................................  8    1   JCK-3
......................Ucggcaucaucaggauucaca.............................................................................................................................................................................................  1    1   JT-1
......................gcggcaucaucCggauucaca.............................................................................................................................................................................................  1    1   LCK-1
......................gcggcaucaucaggauucacC.............................................................................................................................................................................................  1    1   LT-3
......................Ucggcaucaucaggauucaca.............................................................................................................................................................................................  2    1   JCK-3
......................gcggcaucCucaggauucaca.............................................................................................................................................................................................  4    1   LT-2
......................gcggUaucaucaggauucaca.............................................................................................................................................................................................  1    1   JCK-1
```

Mature (gcggcaucaucaggauucaca) / Star (ggaaucuugaugaugcugcau)			
ugaucagucaguauuuucggaugcggcaucaucaggauucacauacuuuauuaaggagagcuuaaauugaguaguaguggaaagaggacgauuucucucuuuugcucgaaagguuccuuucagaugggaaucuugaugaugcugcaucagcaaauauaugacuaugucucccuguuacuucucacauaaauuccaaauauauaaaccccauauauauauauaaaccccauauauauauauauauau			
......................Ucggcaucaucaggauucaca	3	1	JCK-2
......................gcggcaucaucagAauucaca	1	1	JT-1
......................Ccggcaucaucaggauucaca	1	1	JT-1
......................gcggcaucaucCggauucaca	1	1	JCK-3
......................gcNgcaucaucaggauucaca	2	1	LT-2
......................gcggcaucaucaAgauucaca	1	1	LT-2
......................gcggcaucaucaggauucacU	25	1	LCK-2
......................gcggcaucaCcaggauucaca	1	1	LT-2
......................gcggcaucaucCggauucaca	1	1	LT-2
......................gcggcaucaucaggCuucaca	1	1	JCK-2
......................gcgNcaucaucaggauucaca	2	1	JCK-1
......................gcggcaucaucaggauucaca	75	0	LCK-2
......................gcggcaucaucaggCuucaca	1	1	JCK-1
......................gcUgcaucaucaggauucaca	1	1	JCK-2
......................gcggcaucaucaggauucacU	16	1	LCK-3
......................gNggcaucaucaggauucaca	1	1	JCK-3
......................gcgAcaucaucaggauucaca	2	1	JCK-3
......................gcggcaucaucaggauucacU	20	1	LCK-1
......................gcggcaucaucaggauucacC	7	1	JCK-2
......................gNggcaucaucaggauucaca	2	1	JCK-2
......................Acggcaucaucaggauucaca	3	1	JCK-1
......................gcggcaucaucaggauucaca	1309	0	JCK-2
......................gcggcaucaucaggauucacau	1	0	JCK-1
......................gcggcaucaucaggauucacau	1	0	LT-1
......................gcggcaucaucaggauucacUu	4	1	LCK-3
......................gcggcaucaucaggauucacaA	4	1	JCK-2
......................gcggcaucaucaggauucacaC	1	1	LT-3
......................gcggcaucaucaggauucacaC	1	1	JCK-3
......................gcggcaucaucaggauucacau	1	0	LT-3
......................gcggcaucaucaggauucacau	6	0	JCK-3
......................gcggcaucaucaggauucacau	10	0	JCK-2
......................gcggcaucaucaggauucacau	5	0	JT-1
......................gcggcaucaucaggauucacaC	1	1	JCK-2
......................gcggcaucaucaggauucacaA	7	1	LT-2
......................gcggcaucaucaggauucacaA	9	1	JCK-3
......................gcggcaucaucaggauucacaA	5	1	JCK-1
......................gcggcaucaucaggauucacaC	1	1	JCK-1
......................gcggcaucaucaggauucacauU	1	1	LT-1
......................gcggcaucaucaggauucacauU	7	1	LT-3
......................gcggcaucaucaggauucacauU	1	1	LT-2
......................gcggcaucaucaggauucacauU	2	1	LCK-3
......................gcggcaucaucaggauucacauU	1	1	JCK-1
.......................cggcaucaucaggauuca	3	0	LT-3
.......................cggcaucaucaggauuca	1	0	JCK-3
.......................cggcaucaucaggauuca	1	0	JCK-1
.......................cggcaucaucaggauuca	2	0	LT-1
.......................cggcaucaucaggauuca	4	0	LT-2
.......................cggcaucaucaggauuca	4	0	JCK-2
.......................cgNcaucaucaggauuca	1	1	LT-2
.......................cggcaucaucaggauucac	11	0	LT-3
.......................cggcaucaucaggauucac	5	0	LT-1
.......................cggcaucaucaggauucac	7	0	JCK-2
.......................cggcaucaucaggauucac	12	0	JCK-1
.......................cggcaucaucaggauucac	15	0	LCK-3
.......................cggcaucaucaggauucac	4	0	JCK-3
.......................cggcaucaucaggauucaca	15	0	JT-1
.......................cggcaucaucaggauucCca	1	1	LCK-1
.......................cgAcaucaucaggauucaca	1	1	LT-1
.......................cggcaucaucaggauucaca	42	0	LT-1
.......................cggcCucaucaggauucaca	1	1	JCK-2
.......................cggcaucaucaggauucaca	57	0	LT-3
.......................cggcaucaucaggauucacU	1	1	LT-3
.......................cggcaucaucaggauucaca	96	0	JCK-2
.......................cggcaucaucaggauucaca	23	0	LCK-1
.......................cggcaucaucaggauucaca	61	0	JCK-3
.......................cggcaucaucaggauucaca	17	0	LCK-2
.......................cgAcaucaucaggauucaca	1	1	LT-2
.......................cggcaucaucaggauucaca	39	0	LT-2
.......................cggcaucaucaggauucaUa	3	1	LT-2
.......................cggcaucaucaggauucGca	1	1	JCK-1

Mature Star

ugaucagucaguauuuucggaugcggcaucaucaggauucacauacuuuauuaaggagagcuuaaauugaguaguaguggaaagaggacgauuucucucucuuuugcucgaaagguuccuuucagaugggaaucuugaugaugcugcaucagcaaauauaugacuaugucucccuguuacuucucacauaaauuccaaauauauaaaccccauauauauauauaaaccccauauauauauauauauauau

cggcaucaucaggauucaca	32	0	LCK-3
cggcaCcaucaggauucaca	1	1	JCK-3
cggcaucaucaggauucaca	49	0	JCK-1
cggcaucaucaggauucacU	2	1	LT-1
cggcaucaucaggauucacUu	1	1	LT-3
cggcaucaucaggauucacaG	1	1	JCK-1
cggcaucaucaggauucacau	1	0	LT-3
cggcaucaucaggauucacaA	1	1	JCK-1
cggcaucaucaggauucacaA	2	1	JCK-2
cggcaucaucaggauucacaC	3	1	JCK-2
cggcaucaucaggauucacaC	1	1	JCK-3
cggcaucaucaggauucacaG	1	1	JCK-2
cggcaucaucaNgauucacau	1	1	JCK-1
cggcaucaucaggauucacau	14	0	JCK-3
cggcaucaucaggauucacaA	7	1	JCK-3
cggcaucaucaggauucacaC	2	1	JCK-1
cggcaucaucaggauucacau	10	0	JCK-1
cggcaucaucaggauucacau	2	0	LT-1
cggcaucaucaggauucacau	16	0	JCK-2
cNgcaucaucaggauucacau	1	1	JCK-1
cggcaucaucaggauucacauC	3	1	JCK-2
cggcaucaucaggauucacauU	4	1	JCK-1
cggcaucaucaggauucacauU	1	1	LT-3
cggcaucaucaggauucacaua	1	0	JCK-2
cggcaucaucaggauucacauU	6	1	JCK-3
cggcaucaucaggauucacaua	1	0	LT-2
cggcaucaucaggauucacauC	1	1	JCK-3
cggcaucaucaggauucacauU	5	1	JCK-2
ggcaucaucaggauucaca	1	0	JCK-2
ucaggauucacauacuuuauuC	1	1	LT-3
ucaggauucacauacuuuauuU	1	1	JCK-3
aggagagcuuaaauugaguaguagug	3	0	LCK-3
aauugaguaguaguggaaagaggacg	2	0	JCK-3
aauugaguaguaguggaaagaggacg	1	0	JCK-2
ugaguaguaguggaaagaggacg	1	0	JCK-2
aggacgauuucucucucuuuu	1	0	JCK-1
cagaugggaaucuugaugaugc	1	0	LT-3
ugggaaucuugaugaugcug	1	0	LT-2
ugggaaucuugaugaugcugc	2	0	JCK-1
ugggaaucuugaugaugcugc	1	0	JCK-2
ugggaaucuugaugaugcugc	1	0	JCK-3
ugggaaucuugaugaugcugcauc	1	0	JCK-2
ugggaaucuugaugaugcugcauU	1	1	LT-3
ugggaaucuugaugaugcugcauc	1	0	JCK-3
ugggaaucuugaugaugcugcauc	2	0	JCK-1
gggaaucuugaugaugcug	2	0	LT-1
gggaaucuugaugaugcug	1	0	JCK-1
gggaaucuugaugaugcugc	1	0	JCK-2
gggaaucuugaugaugcugc	1	0	LT-3
gggaaucuugaugaugcugca	3	0	JCK-3
gggaaucuugaugaugcugca	5	0	JCK-1
gggaaucuugaugaugcugca	2	0	LT-2
gggaaucuugaugaugcugAa	1	1	LT-1
gggaaucuugaugaugcugca	2	0	JCK-2
gggaaucuugaugaugcugca	2	0	LT-1
gggaaucuugaugaugcugcau	1	0	JCK-3
gggaaucuugaugaugcugcauA	2	1	LT-3
ggaaucuugaugaugcug	2	0	LT-3
ggaaucuugaugaugcugc	7	0	LT-1
ggaaucuugaugaugcugc	5	0	LT-2
ggaaucuugaugaugcugc	9	0	JCK-2
ggaaucuugaugaugcugc	8	0	JCK-1
ggaaucuugaugaugcugc	7	0	JT-3
ggaaucuugaugaugcugU	1	1	LT-3
ggaaucuugaugaugcugc	2	0	JT-2
ggaaucuugaugaugcugc	9	0	JCK-3
ggaaucuugaugaugcugca	1	0	JCK-3
ggaaucuugaugaugcugca	5	0	LT-1
ggaaucuugaugaugcugca	5	0	JT-2
ggaaucuugaugaugcugca	6	0	JT-3

```
                  Mature                                                                                                          Star
ugaucagucaguauuuucggaugcggcaucaucaggauucacauacuuuauuaaggagagcuuaaauugaguaguaguggaaagaggacgauuucucucucuuuugcucgaaagguuccuuucagaugggaaucuugaugaugcugcaucagcaaauauaugacuaugucucccuguuacuucucacauaaauuccaaauauauaaaccccauauauauauauaaaccccauauauauauauauauauau
................................................................................................................................ggaaucuugaugaugcugca......................................................................................................   1   0   LT-3
................................................................................................................................ggaaucuugaugaugcugcau.....................................................................................................   23  0   JCK-2
................................................................................................................................ggaaucuugaugaugcugcau.....................................................................................................   14  0   LT-3
................................................................................................................................ggaaucuugaugaugcugcau.....................................................................................................   15  0   JCK-3
................................................................................................................................ggaaucuugaugaugcugcau.....................................................................................................   9   0   JCK-1
................................................................................................................................ggaaucuugaugaugcugcau.....................................................................................................   7   0   JT-1
................................................................................................................................ggaaucuugaugaugcugcau.....................................................................................................   7   0   LT-2
................................................................................................................................ggaaucuugaugaugcugcau.....................................................................................................   12  0   LT-1
................................................................................................................................ggaaucuugaugCugcugcau.....................................................................................................   1   1   LT-3
.....................................................................................................................................cuugaugaugcugcauca...................................................................................................   1   0   LCK-3
...............................................................................................................................................................augacuaugucucccuguuacu.....................................................................   1   0   JCK-3
...............................................................................................................................................................augacuaugucucccuguuacu.....................................................................   1   0   JCK-1
..................................................................................................................................................................acuaugucucccuguuacuucucaca..............................................................   2   0   JCK-2
..................................................................................................................................................................acuaugucucccuguuacuucucaca..............................................................   1   0   JCK-3
..................................................................................................................................................................acuaugucucccuguuacuucucaca..............................................................   1   0   JCK-1
....................................................................................................................................................................augucucccuAuuacuucuca.................................................................   1   1   LT-2
....................................................................................................................................................................augucucccuguuacuucuca.................................................................   10  0   LT-2
```
